# The complete chloroplast genome sequence of chaya (*Cnidoscolus aconitifolius*) and phylogenetic analysis

**DOI:** 10.1080/23802359.2021.2008829

**Published:** 2022-01-27

**Authors:** Yufeng Qin, Mimi Li, Lianxiang Zhong, Qiulan Wei, Yufei Xiao, Xiaoning Zhang, Zihai Qin

**Affiliations:** a Guangxi Forestry Research Institute, Nanning, China; b Institute of Botany, Jiangsu Province and Chinese Academy of Sciences, Nanjing, China; c The Jiangsu Provincial Platform for Conservation and Utilization of Agricultural Germplasm, Nanjing, China

**Keywords:** Euphorbiaceae, chaya, chloroplast genome, *Cnidoscolus aconitifolius*, plastid

## Abstract

*Cnidoscolus aconitifolius* is a leafy green heathy vegetable and medicinal plant belongs to the family Euphorbiaceae. In the present study, we sequenced the complete chloroplast genome of *C. aconitifolius*, which is 158,658 bp in length and consisted of two copies of inverted repeat (IR) of 26,982 bp separated by a large single copy (LSC) of 87,022 bp and a small single copy (SSC) of 17,672 bp. The GC content of *C. aconitifolius* was 36.3%. A total of 130 genes were predicted, including 86 protein-coding genes, 36 tRNAs and 8 rRNAs. The plastid phylogenomic analysis support *C. aconitifolius* is closely related to *Manihot esculenta*.

*Cnidoscolus aconitifolius* (Mill.) Johnst. (family Euphorbiaceae, subfamily Crotonoideae, tribe Manihoteae), generally known as ‘chaya’ or tree spinach, is a perennial shrub species (Breckon 1979). It is naturally distributed in Mexico and Central America and recently widely introduced into the USA, Europe and China for potential use as a leafy green heathy vegetable and medicinal plant due to its edible and therapeutic impacts (Kuti and Kuti 1999). Chaya has been reported to contain protein, vitamins, calcium, iron and with medicinal benefits especially linked to diabetes mellitus, atherosclerosis, hypertension (Kuti and Torres 1996). However, genetic and genomic information on chaya is scarce. The plastid genomic information reported in this study is valuable for genetic improvement through genetic engineering in chaya.

The fresh mature and healthy leaves of *C. aconitifolius* were sampled at Guangxi Forestry Research Institute, Guangxi Province, China (22°55′30′′N, 108°21′ E) and voucher specimen was saved at Guangxi Forestry Research Institute with collection number 20210317003 (contact: Zihai Qin, 75455621@qq.com). Total genomic DNA was extracted from leaves by modified hexadecyltrimethylammonium bromide (CTAB) method (Doyle and Doyle 1987). A DNA library was prepared using NEB Next^®^ Ultra DNA Library Prep Kit for Illumina (NEB, USA) following manufacturer’s instructions with an insert size of 300 bp and subsequently sequenced on a Illumina Hiseq X-ten platform (San Diego, USA) at Novogene Co., Ltd. (Beijing, China). The Paired-end (PE) reads were directedly assembled into plastid genome using NOVOPlasty 4.3.1 (Dierckxsens et al. 2017) with default parameters using *Manihot esculenta* (NC_010433) as a reference. The genome annotation was performed by GeSeq (Tillich et al. 2017) and adjusted by manual in Geneious 11.1.5 (Kearse et al. 2012). The complete plastid sequence was deposited to the National Center for Biotechnology Information (NCBI) under accession number MZ045411.

The plastid genome of *C. aconitifolius* was 158,658 bp in length and consisted of two copies of IR of 26,982 bp separated by a LSC of 87,022 bp and a SSC of 17,672 bp. The total GC content of *C. aconitifolius* was 36.3%, of which was 33.9%, 30.3%, and 42.2% in LSC, SSC and IRs regions, respectively. A total of 130 genes were predicted, including 86 protein-coding genes (PCGs), 36 transfer RNA genes (tRNA) and 8 ribosomal RNA genes (rRNA). Seven PCGs (*rpo*C1, *ndh*B, *ndh*A, *pet*D, *rps*16, *pet*B, *rpl*2) and five tRNA (*trn*K-UUU, *trn*I-GAU, *trn*A-UGC, *Trn*V-UAC, *trn*L-UAA) had one intron, while two PCGs (*clp*P and *ycf*3) had two introns.

A total of 12 plastomes were used for the phylogenomic analysis. Two species of *Euphorbia* – *E. lathyris* (MT241376) and *E. smithii* (MN646684) – were defined as outgroups. The complete plastid sequences were aligned using MAFFT 7.409 (Katoh and Standley 2013). The plastid phylogenomic analysis was generated by maximum likelihood (ML) in RAxML with 1000 bootstrap replicates (Stamatakis 2014) under the GTR + G model. Our phylogenetic analyses revealed that the tribe Manihoteae is a monophyletic clade (bootstrap support 100%) that is sister to the tribe Micrandreae (Figure 1). The result is similar with the previous work reported by Wurdack et al. (2005) based on the plastid *rbc*L and *trn*L-F sequences indicating that the cp genome is a powerful tool to resolve the deep phylogenetic relationships within the family Euphorbiaceae. Within the tribe Manihoteae, *C. aconitifolius* was most closely related to *Manihot esculenta*.

**Figure 1.**
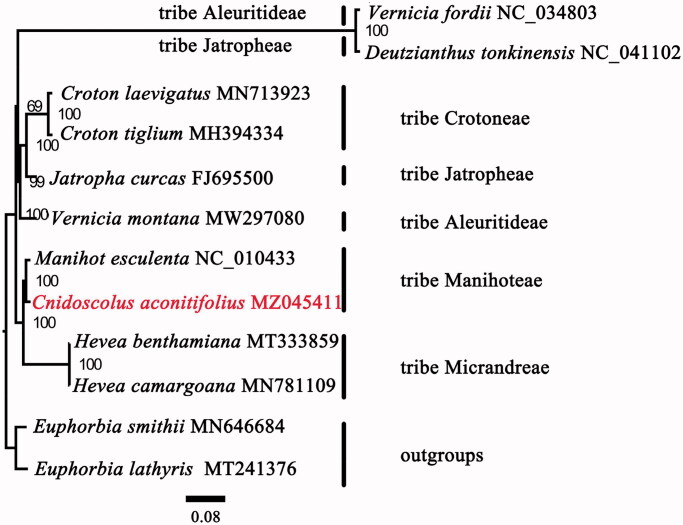
Maximum likelihood phylogenetic tree of *Cnidoscolus aconitifolius* with 1000 bootstrap replicates.

## Data Availability

The genome sequence data that support the findings of this study are openly available in GenBank of NCBI at (https://www.ncbi.nlm.nih.gov/) under the accession no. MZ045411. The associated BioProject, SRA, and Bio-Sample numbers are PRJNA743565, SRR15036486 and SAMN20055435, respectively.
